# Sirt3 Pharmacologically Promotes Insulin Sensitivity through PI3/AKT/mTOR and Their Downstream Pathway in Adipocytes

**DOI:** 10.3390/ijms23073740

**Published:** 2022-03-29

**Authors:** Alexandra Yatine Lee, Sabrina Marie Christensen, Nhi Duong, Quoc-Anh Tran, Hou Mai Xiong, Jennifer Huang, Sarah James, Dimple Vallabh, George Talbott, Melanie Rose, Linh Ho

**Affiliations:** 1 College of Medicine, California Northstate University, Elk Grove, CA 95757, USA; alexandra.lee6556@cnsu.edu (A.Y.L.); sabrina.christensen6251@cnsu.edu (S.M.C.); 2 College of Pharmacy, California Northstate University, Elk Grove, CA 95757, USA; nhi.duong9826@cnsu.edu (N.D.); quoc-anh.tran6131@cnsu.edu (Q.-A.T.); hou.xiong1239@cnsu.edu (H.M.X.); jennifer.huang7732@cnsu.edu (J.H.); sarah.james5624@cnsu.edu (S.J.); dimple.vallabh8822@cnsu.edu (D.V.); george.talbott@cnsu.edu (G.T.); melanie.rose@cnsu.edu (M.R.)

**Keywords:** Sirt3, Sirt3 activator, Sirt3 inhibitor, honokiol, 3-TYP, adipocytes, mitochondrial sirtuins, insulin pathway, insulin/IGF-1 (Insulin-like Growth Factor-1) signaling pathway, PI3K/AKT/mTOR signaling pathway

## Abstract

Sirtuin-3 (Sirt3) is a major mitochondrial deacetylase enzyme that regulates multiple metabolic pathways, and its expression is decreased in diabetes type 1 and type 2 diabetes. This study aimed to elucidate Sirt3′s molecular mechanism in regulating insulin sensitivity in adipocytes that can contribute to the effort of targeting Sirt3 for the treatment of obesity and type 2 diabetes. We found that the Sirt3 activator honokiol (HNK) induced adipogenesis compared to the control, in contrast to Sirt3 inhibitor, 3-TYP. Accordingly, HNK increased expression of adipocyte gene markers, gene-involved lipolysis and glucose transport (GLUT4), while 3-TYP reduced expression of those genes. Interestingly, 3-TYP caused an increase in gene expression of adipocyte-specific cytokines including IL6, resistin, and TNF-α. However, changes in adipocyte-specific cytokines in HNK treated cells were not significant. In addition, HNK stimulated insulin pathway by promoting insulin receptor beta (IRβ) and PI3K/AKT/mTOR pathways, resulting in an increase in phosphorylation of the forkhead family FoxO1/FoxO3a/FoxO4 and glycogen synthase kinase-3 (GSK-3β), opposing 3-TYP. In line with these findings, HNK increased free fatty acid and glucose uptake, contrary to 3-TYP. In conclusion, Sirt3 activator-HNK induced adipogenesis and lipolysis reduced adipocytes specific cytokines. Intriguingly, HNK activated insulin signaling pathway and increased free fatty acid as well as glucose uptake and transport, in sharp contrast to 3-TYP. These results indicate that, via insulin signaling regulation, Sirt3 activation by HNK improves insulin resistance, while Sirt3 inhibition by 3-TYP might precipitate insulin resistance.

## 1. Introduction

Type 2 diabetes mellitus (T2DM) is the seventh leading cause of death in the United States, and about 10% of the global population have been affected by this disease [1,2]. T2DM or insulin resistance is characterized by high glucose level in the blood because of a decreased insulin sensitivity in metabolic tissues, leading to complications including obesity, hypertension, atherosclerosis, liver failure, and certain cancers [3]. It is a chronic metabolic abnormality tightly associated with obesity [4]. Obesity is characterized by the abnormal expansion of adipose tissue and the dysregulated production of adipose tissue-secreted proteins (adipokines) and lipids, low-grade inflammation, and accumulation of extracellular matrix (ECM) [4,5,6,7]. Adipose tissue is one of the major metabolic target tissues of insulin, and disruptions in glucose uptake in adipose tissue is associated with insulin resistance [8].

Integrating local and systemic signals with proper mitochondrial function is critical for maintaining metabolic homeostasis. Mitochondrial defects and mitochondrial decline have been observed in metabolic disorders, age-related metabolic and degenerative diseases, aging, and cancer [9,10]. Mitochondrial dysfunction in various tissues has been implicated in the pathology of T2DM and metabolic syndrome. Reduced mitochondrial oxidative capacity, together with increased reactive oxygen species (ROS) generation, can increase insulin resistance [10].

Adipocytes play a crucial role in glucose and lipid homeostasis, and dysfunction in these cells is linked to insulin resistance and, therefore, type 2 diabetes [11]. Adipokines, which are released from adipocytes, are involved in metabolic control and play a regulatory role in diabetes and obesity [12]. Both disease processes are precipitated by insulin resistance, which stems from increased serum fatty acids and altered secretion of these adipokines [12]. Such altered secretions of serum adipokines can lead to increased oxidative stress, which may induce hyperglycemic crises in diabetic patients [13]. The mitochondrial deacetylase enzyme Sirt3 has been found to decrease such oxidative stress and abnormal fatty acid oxidation, two processes that have both been implicated in diabetes and obesity [14]. 

Sirt3 has been identified as a major mitochondrial deacetylase enzyme and highly expressed in metabolic tissues including liver, kidney, skeletal muscle, brown adipose tissue, heart, and brain [15,16,17,18]. Under fasting, Sirt3 is upregulated in the liver and brown adipose tissue [19]. In contrast to fasting, long-term high-fat feeding has been shown to downregulate levels of Sirt3 mRNA, leading to hyperacetylation and decreased activity of mitochondrial proteins [11,12] involved in oxidative metabolism [20,21]. Sirt3 knockout mice fed a high fat diet showed accelerated obesity, metabolic syndrome, glucose intolerance, and insulin resistance. Patients with a loss-of-function mutation in Sirt3 have increased susceptibility to the development of the metabolic syndrome [20]. These findings point to the important role Sirt3 plays in maintaining metabolic homeostasis and the hypothesis that Sirt3 might be a potential target for the treatment of type 2 diabetes. 

The protective effects of Sirt3 within the setting of oxidative stress can be enhanced with the addition of natural, plant-derived Honokiol. A lignan from the Magnolia genus, Honokiol, with its ability to target free radicals [22] and restore antioxidants [23], has been identified as a powerful antioxidant and, therefore, enhancer of Sirt3′s inherent anti-oxidative properties. In contrast, 3-TYP (3-(1H-1,2,3-triazol-4-yl) pyridine) is a Sirt3 inhibitor that decreases the protective effects of certain proteins on oxidative stress, more specifically pathways related to superoxide dismutase (SOD) [24].

In this study, we identified whether the Sirt3 activator honokiol improves insulin sensitivity in adipocytes in contrast with another Sirt3 inhibitor, 3-TYP. We investigated the effects of Sirt3 modulators on the expression of proteins that are crucial to the insulin PI3/AKT/mTOR signaling pathway in adipocytes. We hope that our findings elucidate the molecular mechanism of Sirt3 modulators in regulating insulin signaling pathway and highlight the therapeutic potential of Sirt3 activator in the treatment of type 2 diabetes and metabolic diseases. 

## 2. Results

### 2.1. Sirt3 Activator-Honokiol Enhances Adipocyte Differentiation in 3T3-L1 Preadipocytes. In Contrast, Sirt3-Inhibitor-3-TYP Inhibits Adipogenesis of 3T3-L1 Preadipocytes

**Figure 1:**
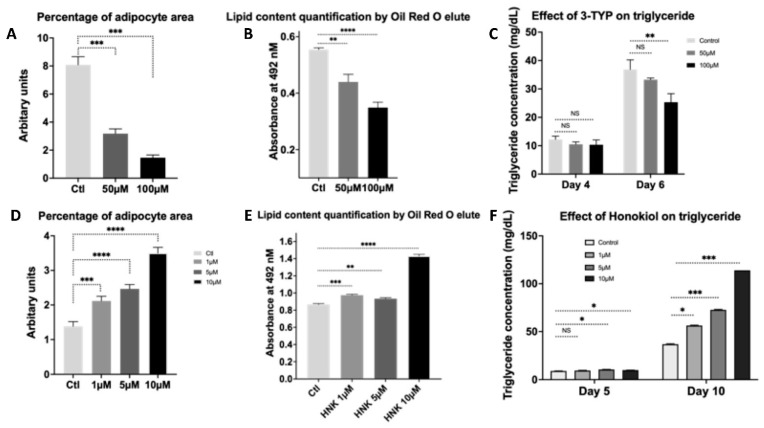
HNK enhances differentiation in 3T3-L1 preadipocytes into adipocytes. In contrast, 3-TYP decreased adipogenesis. (**A**) Measurement of percentage of adipocyte area. Thirty images of each condition were acquired at 250×, and blinded analyses were performed using ImageJ-analysis software; (**B**) lipid content quantification of isopropanol elutes from ORO staining; and (**C**) triglyceride measurement of 3T3-L1 cells treated with 3-TYP at concentrations of 50 µM, 100 µM, and untreated as control. (**C**) Measurement of percentage of adipocyte area. Thirty images of each condition were acquired at 250×, and blinded analyses were performed using ImageJ-analysis software; (**D**) lipid content quantification of isopropanol elutes from ORO staining; and (**E**) triglyceride measurement of 3T3-L1 cells treated with Honokiol at concentrations of 1, 5, and 10 µM versus controls. Two samples for each treatment versus control and data were collected from at least three independent experiments. Statistical analysis was performed using two-tailed unpaired Student’s *t* test (**A**,**C**,**D**,**F**) and two-way analysis of variance analysis (ANOVA) followed by post hoc Dunnette’s multiple comparisons (**B**,**E**), ns: not significant; * *p* < 0.05; ** *p* < 0.01, *** *p* < 0.001, **** *p* < 0.0001.

Sirt3-knockout mice fed a high fat diet showed accelerated obesity, metabolic syndrome, glucose intolerance, and insulin resistance. Patients with a loss-of-function mutation in Sirt3 have increased susceptibility to the development of the metabolic syndrome [20]. Dysregulation of adipocyte proliferation and differentiation causes obesity or lipoatrophy, cardiovascular diseases, and diabetes [25,26]. Recently, Sirt3 was found to induce adipocyte differentiation [27]. To determine whether HNK as a Sirt3 activator and 3-TYP as Sirt3 inhibitor cause an effect on adipogenesis, 3T3-L1 preadipocytes (ATCC, CL173) were used for differentiating into adipocytes. 3T3-L1 cells were treated with HNK at 1, 5, and 10 µM or with 3-TYP at 50 µM and 100 µM, or untreated as control and differentiated into adipocytes by the adipocyte differentiation procedure described. HNK increased adipogenesis compared to the control by analyzing Oil Red O staining and measuring isopropanol elution to quantify lipid content. In contrast, 3-TYP decreased adipogenesis compared to control in 3T3-L1 cells (Figure 1).

### 2.2. Honokiol Enhances the Expression of Adipocyte-Specific and Adipogenesis Genes. In Contrast, 3-TYP Decreases Levels of the Expression of Adipocyte-Specific and Adipogenesis Genes

**Figure 2:**
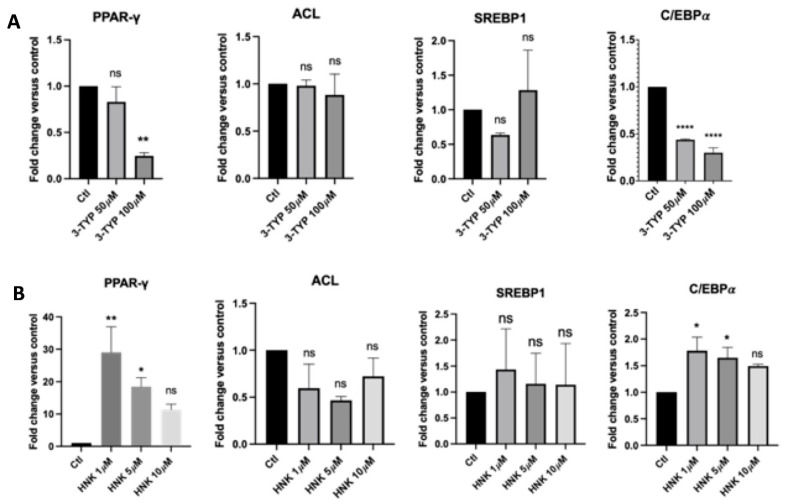
The effect of HNK and 3-TYP on adipocyte-specific and adipogenesis genes. (**A**) Effect of HNK at varying concentrations on gene expression of PPARγ, ACL, SREBP1, and C/EBPα versus controls; (**B**) Effect of 3-TYP at varying concentrations on gene expression of PPARγ, ACL, SREBP1, and C/EBPα versus controls. Each sample was run in triplicate and data were collected from at least three independent experiments. Statistical analysis was performed by one way ANOVA with Dunnett’s multiple comparisons test, ns: not significant, * *p* < 0.05; ** *p* < 0.01; **** *p* < 0.0001.

To determine whether induction or inhibition of adipogenesis by HNK or 3-TYP, respectively, is associated with alterations in expression levels of adipocyte marker genes, we measured expression levels of peroxisome proliferator-activated receptor gamma (PPARγ), ATP citrate lyase (ACL), sterol regulatory element- binding protein 1 (SREBP1), and CCAAT/enhancer binding protein (C/EBPα) using quantitative polymerase chain reaction (qPCR). HNK increased gene expression of adipocyte gene markers, especially PPARγ and C/EBPα significantly (Figure 2B). In opposition to HNK, 3-TYP decreased PPARγ and C/EBPα significantly, especially at 100 µM concentration (Figure 2A). However, both HNK and 3-TYP did not significantly change ACL and SREBP1 gene expression (Figure 2). 

### 2.3. Honokiol Enhances the Expression of Genes in Lipolysis and Glucose Transport (GLUT4). In Contrast, 3-TYP Decreases Levels of the Expression of Genes in Lipolysis and Glucose Transport (GLUT4)

**Figure 3:**
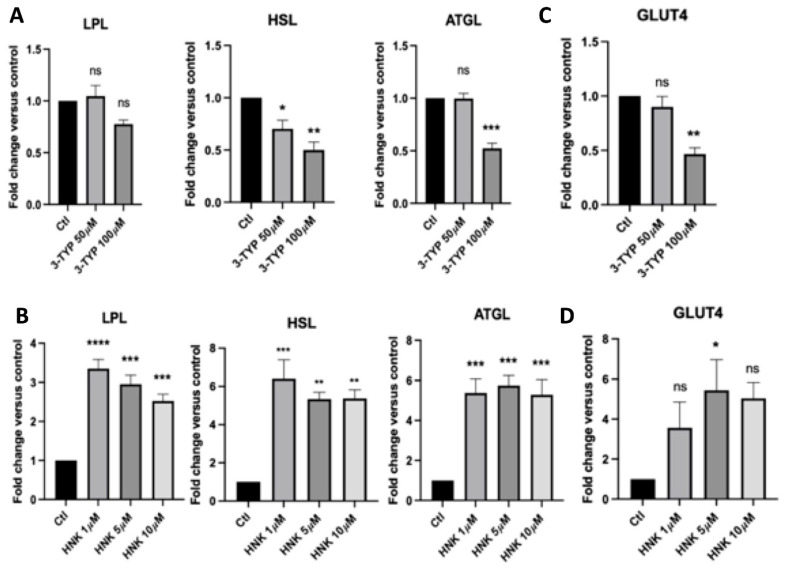
The effect of 3-TYP and HNK on gene expressions in lipolysis and glucose transport in adipocytes. (**A**,**C**) Effect of 3-TYP at varying concentrations and (**B**,**D**) effect of HNK at varying concentrations on gene expression of LPL, HSL, and ATGL in lipolysis and GLUT4 in glucose transport in adipocytes versus controls. Each sample was run in triplicate and data were collected from at least three independent experiments. Statistical analysis was performed using one-way ANOVA followed by Dunnett’s multiple comparisons test, ns: not significant, * *p* < 0.05; ** *p* < 0.01, *** *p* < 0.001, **** *p* < 0.0001.

We also identify whether HNK and 3-TYP can affect lipolysis by using qPCR to assess the gene expression levels of lipolysis makers, including lipoprotein lipase (LPL), adipose triglyceride lipase (ATGL), and hormone-sensitive lipase (HSL), as well as glucose transport (GLUT4). In the cells treated with HNK, we observed a significant increase in the expression of genes in lipolysis, such as LPL, HSL, ATGL, and glucose transport as compared to the control (Figure 3B). There was an increased trend in GLUT4 expression and at 5 µM of HNK, GLUT4 was significantly elevated compared to the control (Figure 3D). In contrast to HNK, 3-TYP decreased expression of genes involved in lipolysis and glucose transport GLUT4 (Figure 3A,C), especially at 100 µM. LPL expression level was decreased at 100 µM of 3-TYP; however, it was not significant (Figure 3A).

### 2.4. Honokiol Suppressed Adipocyte-Specific Cytokines. In Contrast, 3-TYP Increased Adipocyte-Specific Hormones

**Figure 4:**
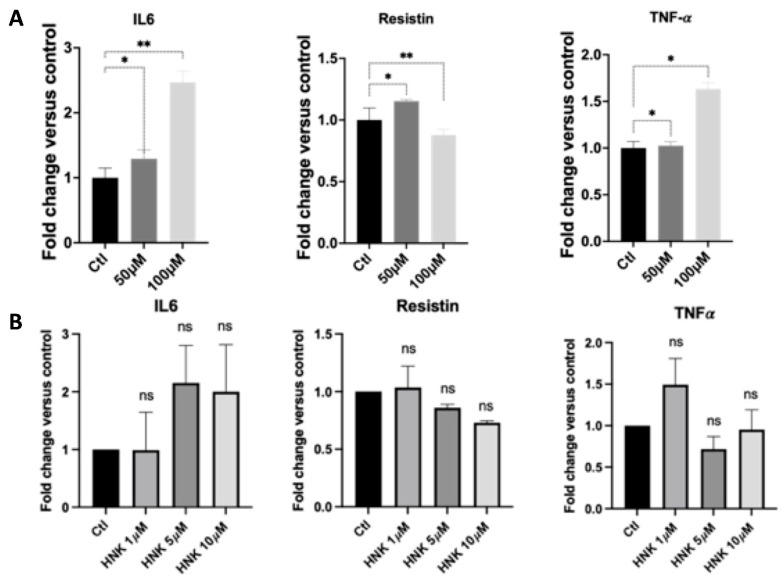
The effect of HNK and 3-TYP on adipocyte-specific cytokines (IL-6, resistin, and TNF-α) in 3T3-L1 adipocytes. (**A**) Effect of 3-TYP; and (**B**) effect of HNK on gene expression of IL-6, resistin, and TNF-α genes. Each sample was run in triplicate and data were collected from at least three independent experiments. Statistical analysis was performed using one-way ANOVA followed by Dunnett’s multiple comparisons test, ns: not significant, * *p* < 0.05; ** *p* < 0.01.

Adipocytes have been characterized to release several adipokines or cytokines that work locally in an autocrine and paracrine fashion or peripherally in an endocrine fashion [28]. Adipocyte hypertrophy occurs during obesity, causing dysregulation of the microenvironment within adipose depots and systemically alters peripheral tissue metabolism [28]. The dysregulation of adipokines has been implicated in obesity, type 2 diabetes, and cardiovascular disease [29]. To determine whether HNK and 3-TYP-treated adipocytes cause any change in adipocyte-specific cytokine expression, we measured expression of these cytokines by qPCR. Interestingly, 3-TYP caused an increase in gene expression of adipocyte-specific cytokines, including IL6, resistin, and TNF-α, especially at 100 µM concentration. However, resistin was decreased at 100 µM concentration of 3-TYP. Intriguingly, changes in adipocyte-specific cytokines in HNK-treated cells were not significant (Figure 4).

### 2.5. Honokiol Promotes the PI3K/AKT Pathway to Enhance Insulin Signaling in 3T3-L1 Adipocytes. In Contrast, 3-TYP Inhibits the PI3K/AKT Pathway to Suppress Insulin Signaling in 3T3-L1 Adipocytes

**Figure 5:**
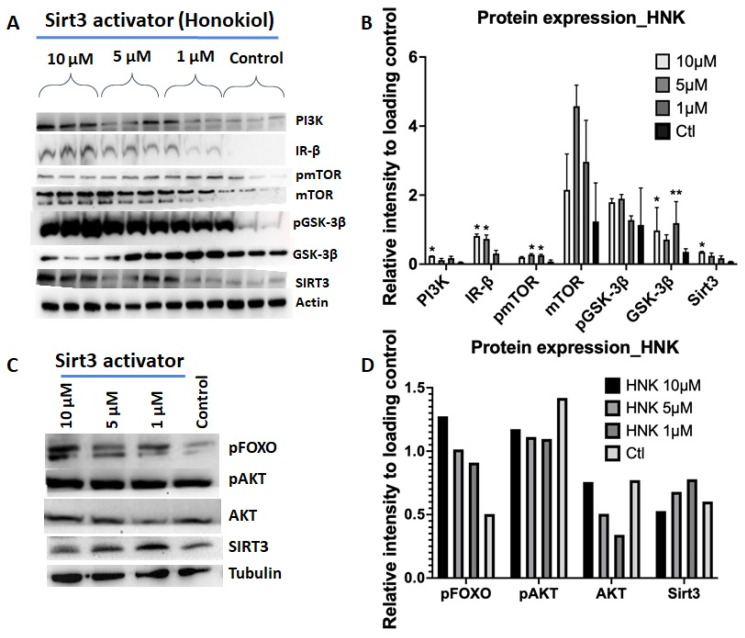

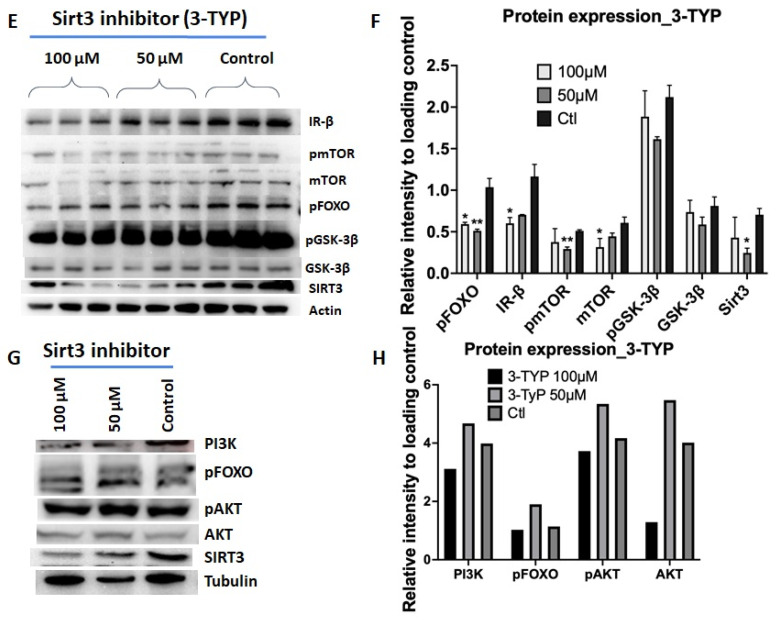
HNK activated PI3/AKT/mTOR to promoting insulin signaling pathway; meanwhile, 3-TYP inhibited PI3/AKT/mTOR to suppress insulin signaling pathway. WB and quantification of protein band intensity of 3T3-L1 adipocytes treated (**A**–**D**) with HNK or (**E**–**H**) with 3-TYP at various concentrations compared to controls using antibodies in insulin signaling pathway. Tubulin and actin were used as loading control and Sirt3 as a reference. (**A**) WB and quantification of protein intensity of 3T3-L1 adipocytes treated with HNK at 1, 5, and 10 µM and control against (**A**,**B**) PI3K, IR-β, mTOR, pmTOR, GSK-3β, pGSK-3β and (**C**,**D**) pFOXO, AKT, pAKT antibodies; (**E**,**F**) WB of 3T3-L1 adipocytes treated with 3-TYP at 50 µM and 100 µM and control against IR-β, mTOR, pmTOR, GSK-3β, pGSK-3β and (**G**,**H**) PI3K, pFOXO, AKT, pAKT antibodies. Actin as loading controls and Sirt3 as a reference. Data collected from at least three independent experiments. Statistical analysis was performed using two-way ANOVA followed by Dunnett’s multiple comparisons test, * *p* < 0.05; ** *p* < 0.01.

Insulin signaling through the PI3K/AKT/mTOR pathways is considered to have an important role in insulin resistance. Therefore, we examined the effects of HNK and 3-TYP on this insulin signaling pathways in 3T3-L1 adipocytes. In HNK-treated cells, we observed an increase in protein expression of IR-β, PI3K, and mTOR, as well as phosphorylation level of the forkhead family FoxO1/FoxO3a/FoxO4 and glycogen synthase kinase-3 (GSK-3β), protein kinase B (AKT), and mammalian target of rapamycin (mTOR) (Figure 5A–D). These proteins have a function in insulin signaling pathways. In an opposite manner, 3-TYP treated cells showed decreased expression and phosphorylation of these proteins in the insulin signaling pathway (Figure 5E–H). These results indicate that honokiol induces PI3/AKT/mTOR insulin cascade pathway in adipocytes. In sharp contrast, 3-TYP inhibits this insulin signaling pathway. 

### 2.6. Glucose Uptake in HNK and 3-TYP Treated 3T3-L1 Adipocytes

**Figure 6:**
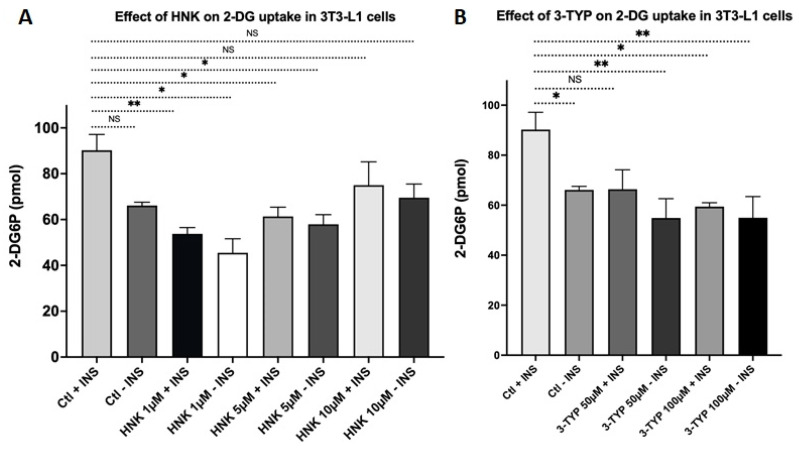
Glucose uptakes in 3T3-L1 adipocytes treated with HNK or 3-TYP. Glucose uptake in the presence or absence of 1 µM of insulin in 3T3-L1 adipocytes treated with (**A**) 3-TYP at concentrations of 50 and 100 µM; or (**B**) HNK at 1, 5, and 10 µM compared to untreated cells. Data was collected from at least three independent experiments. Statistical analysis was performed using two-way ANOVA followed by Dunnett’s multiple comparisons test, NS: not significant, * *p* < 0.05; ** *p* < 0.01.

We identified that HNK, counter to 3-TYP, induced adipogenesis and adipocyte-specific and adipogenesis genes. We next examined whether Sirt3 induction by HNK and Sirt3 reduction by 3-TYP have an effect on insulin sensitivity. We measured glucose uptake of cells treated with HNK or 3-TYP compared to the untreated controls. HNK increased glucose uptake in 3T3-L1 adipocytes at 10 µM with and without insulin, although this was not significant compared to the control without insulin (Figure 6A). HNK with insulin did not bring up the glucose uptake level as high as in the control with insulin (Figure 6A). 3-TYP significantly inhibited glucose uptake in 3T3-L1 adipocytes at 100 µM compared to control in the presence of insulin (Figure 6B). 

### 2.7. Sirt3 Induction Enhanced Free Fatty Acid Uptake, Meanwhile Sirt3 Inhibition Inhibited Free Fatty Acid Uptake in 3T3-L1 Adipocytes

**Figure 7:**
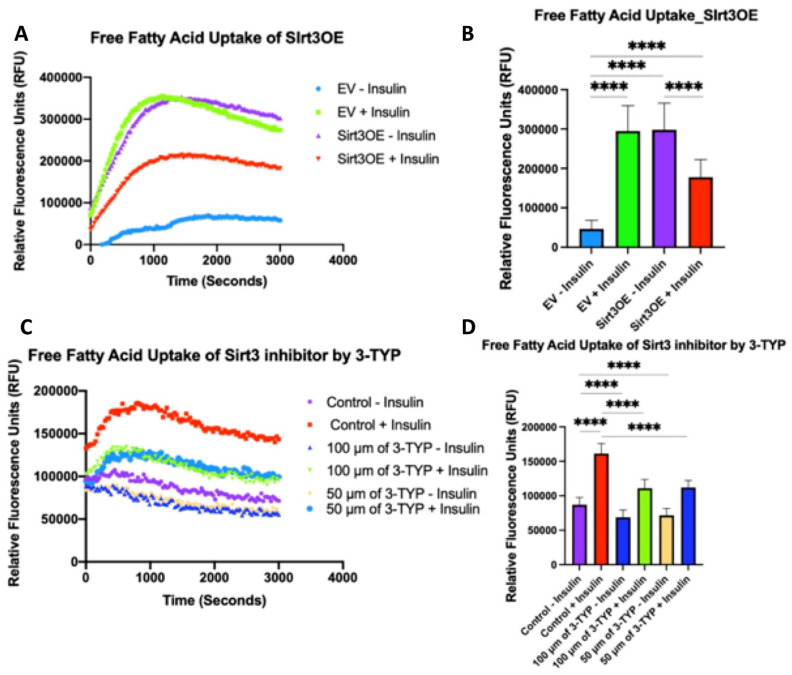
Sirt3 induction increased free fatty acid uptakes. Meanwhile, Sirt3 inhibition by 3-TYP inhibited free fatty acid uptake in 3T3-L1 adipocytes. Free fatty acid uptake for Sirt3 overexpression cells +/− insulin (**A**) shown in XY correlation graph and (**B**) in bar graphs (with the reading at 60 min) of relative fluorescence units versus time points with kinetic reading. Free fatty acid uptake assays were performed for cells treated with Sirt3 inhibitor 3TYP +/− insulin (**C**) shown in XY correlation graph and (**D**) in bar graphs (with the reading at 60 min) of relative fluorescence units versus time points with kinetic reading. Data was collected from at least three independent experiments. Statistical analysis was performed using one-way ANOVA followed by Tukey’s multiple comparisons test, **** *p* < 0.0001.

We next examined whether Sirt3 induction and Sirt3 reduction have an effect on insulin sensitivity by measuring free fatty acids uptake of 3T3-L1 adipocytes. Cells that overexpressed Sirt3 led to a significant increase in uptake of free fatty acids compared to the empty vector with or without insulin. The free fatty acid (FFA) uptake in Sirt3OE cells without insulin was higher compared to Sirt3OE cells with insulin. Contrary to HNK, 3-TYP treated 3T3-L1 adipocytes showed a remarkable decrease in free fatty acid uptake (Figure 7). 

## 3. Discussion

Obesity has been correlated with insulin resistance and seen as a major risk factor for the development of type 2 diabetes [30]. Adipose tissue is one of the major metabolic target tissues of insulin, and disruptions in glucose uptake in adipose tissue are associated with insulin resistance [8]. Obesity is characterized by increased adipose tissue mass, which is driven either by increased adipocyte size (hypertrophy) or increased adipogenesis (hyperplasia) [31]. Adipogenesis is the process in which fibroblast-like progenitor cells restrict their fate to the adipocyte lineage-forming preadipocytes (commitment), then undergo differentiation, growth arrest, and accumulate lipids and become functional, insulin-responsive mature adipocytes. When the committed preadipocyte arrests its growth, it activates the master regulator of adipogenesis peroxisome proliferator-activated receptor-γ (PPARγ) and transcription co-activators CCAAT/enhancer binding protein α and β (C/EBPα and C/EBPβ) [32,33,34]. Lipid accumulation drives the expression of early adipocyte markers such as the adipocyte fatty acid-binding protein (AP2) and the insulin-sensitive transporter GLUT4 [35]. Mature adipocytes express all the markers of early adipocyte differentiation as well as the adipokines adiponectin and leptin, the lipases adipose triglyceride lipase (ATGL) and lipoprotein lipase (LPL), and high levels of the lipid-droplet-associated protein perilipin 1 [36,37,38]. Using 3T3-L1 preadipocytes to differentiate them into adipocytes and treat them with Sirt3 activator HNK or Sirt3 inhibitor 3-TYP, we found that Sirt3 induction by HNK led to an increase in adipogenesis and triglycerides. In contrast, adipogenesis and triglyceride levels were reduced by the Sirt3 inhibitor 3-TYP in comparison to the controls. This result was consistent with our previous studies showing that Sirt3 induced adipocyte differentiation from bone marrow-derived stromal ST2 cells [27], and that Sirt3 is involved in inducing the differentiation of bone marrow stromal cells into different cell lineages [39].

In accordance with increased adipogenesis by Sirt3 activator HNK, we showed that this adipogenesis induction was associated with increased expression of adipocyte gene markers, especially PPARγ and C/EBPα. Conversely, expression of adipocyte-specific genes was decreased in Sirt3 inhibitor 3-TYP treated 3T3-L1 adipocytes. The expansion of adipose depots characterizes obesity results from increased numbers of individual adipocytes (hyperplasia) and from the hypertrophy of adipocytes [40,41]. Importantly, there exists a broad individual variation in the size and expandability of different adipose tissue depots in humans. This factor is crucially important in understanding the relationship between obesity and insulin resistance, as expansion of some depots is associated with increased risk, whereas expansion of others is associated with decreased risk [42,43]. In fact, in obese adolescents a high ratio of visceral to subcutaneous fat is associated with impaired adipogenesis/lipogenesis, as assessed by gene expression, and a low ratio of visceral to subcutaneous fat is not associated with increased insulin sensitivity [44]. Critically, the enhanced adipogenesis, inferred by the presence of hyperplasia in subcutaneous adipose tissue, correlates with decreased risk of glucose and insulin abnormalities [45]. Taken together, our results show that pharmacologic activation of Sirt3 by HNK increased adipogenesis, suggesting Sirt3 inducers to be good candidates for improving insulin sensitivity and decreasing the risk of insulin resistance. 

In addition, we found that Sirt3 induction by HNK enhanced the expression genes in lipolysis, including LPL, HSL, ACL, ATGL and glucose transport (GLUT4), while Sirt3 inhibition showed a decrease in expression of those genes. Lipolysis in adipocytes is the hydrolysis of triacylglycerides (TAG) to generate fatty acids and glycerol under fasting conditions or elevated energy demands. Lipolysis is known to be regulated by adrenergic activation and insulin-mediated control, resulting in the activation lipases HSL and ATGL as well as phosphorylation of perilipin by protein kinase A [46]. In obese and insulin-resistant people, lipolysis induced by catecholamines is reduced in subcutaneous adipose tissue [47]. Natriuretic peptides-induced lipolysis is also decreased in the obese state [48]. It has been found that lipoprotein lipase activity in adipose tissue is stimulated by insulin. In our findings, Sirt3 induction by HNK could enhance lipolysis via increasing expression of genes that are involved in lipolytic activity, suggesting HNK improved insulin activity. GLUT4 is the insulin-responsive facilitative glucose transporter expressed in adipose, skeletal muscle, and cardiac muscle tissues. GLUT4 expression levels are associated with whole-body insulin-mediated glucose homeostasis [49,50], as well as glucose and lipid homeostasis [51]. In addition, insulin-resistant glucose transport in adipocytes from obese and diabetic subjects correlates with reduced GLUT4 mRNA and protein expression [52,53,54], confirming a role of GLUT4 for insulin-dependent glucose homeostasis. Furthermore, it has been reported that GLUT4 expression decreased due to insulin resistance and high levels of inflammatory markers in rats with metabolic syndrome [55]. We demonstrated that the Sirt3 activator HNK increased glucose transport *GLUT4* gene expression while Sirt3 inhibitor 3-TYP decreased its expression. This result implies that pharmacological strategies to activate Sirt3 might have beneficial effects in diabetic states via enhancing GLUT4 expression. Taken together, these findings indicate the significant role of Sirt3 in the regulation of metabolic activity. Inhibition of Sirt3 leads to a downregulation of lipolysis and glucose transport, suggesting that dysfunctional adipocytes may have lower levels of Sirt3 compared to normal adipocytes. Activation of Sirt3 by its activator, HNK, improves insulin sensitivity via enhancing lipolysis and glucose transport, which might be beneficial for treatment of insulin resistance or type 2 diabetes. 

Adipocytes hare known to release several cytokines that work locally in an autocrine and paracrine fashion or peripherally in an endocrine fashion [28]. Adipocyte hypertrophy occurs during obesity, causing dysregulation of the microenvironment within adipose depots and systemically altering peripheral tissue metabolism [28]. The dysregulation of cytokines has been implicated in obesity, type 2 diabetes, and cardiovascular disease [29]. Several types of pro-inflammatory adipokines are secreted by obese adipose tissues such as interleukin 6 (IL-6), resistin, and tumor necrosis factor α (TNF-α) [56]. Interestingly, our data shows that Sirt3 inhibitor 3-TYP caused an increase in gene expression of adipocyte-specific cytokines including IL6, resistin (decreased at 100 µM concentration of 3-TYP), and TNF-α; however, changes in adipocyte-specific cytokines in HNK treated cells were not significant. This suggests that insulin resistance might have a low level of Sirt3 expression and Sirt3 activator, and that HNK has beneficial effects on restoring dysregulation of proinflammatory cytokines in metabolic abnormalities. Increasing systemic TNF-α and IL-6 in obesity might impair insulin signaling pathway [40].

Indeed, we found that the Sirt3 activator HNK stimulated insulin pathway via promoting insulin receptor beta (IRβ) and PI3K/AKT/mTOR pathways, resulting in an increase in phosphorylation of the forkhead family FoxO1/FoxO3a/FoxO4 and glycogen synthase kinase-3 (GSK-3β). Contrary to HNK, Sirt3 inhibition by 3-TYP suppressed insulin pathway by inhibiting insulin receptor beta (IRβ) and PI3K/AKT/mTOR pathway, resulting in a decrease in phosphorylation of the forkhead family FoxO1/FoxO3a/FoxO4 and glycogen synthase kinase-3 (GSK-3β). In line with these findings, Sirt3 activation increased free fatty acid and glucose uptake, as opposed to Sirt3 inhibition.

By using contrast models of Sirt3 activator versus Sirt3 inhibitor in 3T3-L1 cells, our study found that Sirt3 activation stimulates adipogenesis, lipolysis, and glucose transport in contrast to Sirt3 inhibition. These findings emphasize the mechanisms that control the expandability of adipose tissue, including its high capacity for adipocyte differentiation, and suggest lipid storage may be key factors in determining diabetes risk in obesity. These data also confirm that increased adipogenesis via Sirt3 inducer is associated with decreased risk of insulin resistance and improved insulin sensitivity, as opposed to Sir3 inhibitor. In addition, Sirt3 induction promotes insulin signaling through activating PI3K/AKT/mTOR pathway and subsequently enhances free fatty acid and glucose uptake. In sharp contrast, Sirt3 inhibition inhibits insulin signaling by suppressing PI3K/AKT/mTOR pathway and subsequently decreases free fatty acid and glucose uptake. It might be interesting to investigate Sirt3 activator versus Sirt3 inhibitor in different adipose tissue depots in an animal model. The data suggest that Sirt3 activator might be a promising candidate for treatment of obese-induced insulin resistance and metabolic diseases, while Sirt3 inhibition might result in insulin resistance and other metabolic dysregulations. 

## 4. Materials and Methods

### 4.1. Sirt3 Activator Honokiol and Sirt3 Inhibitor 3-TYP Treatments

3T3-L1 cells were treated with the Sirt3 inhibitor 3-TYP (3-(1H-1,2,3-triazol-4-yl) pyridine) (Selleckchem, Houston, TX, USA) at 50 µM and 100 µM, or with the Sirt3 activator Honokiol (Tocris, Minneapolis, MN 55413, USA) at 1, 5, and 10 µM or were untreated as control and differentiated into adipocytes by the adipocyte differentiation procedure described below. Honokiol and 3-TYP treatments were applied during the process of differentiation of adipocytes (day 1 to day 6).

### 4.2. Transfections

For SIRT3 overexpression (Sirt3OE), 3T3-L1 cells were transfected with plasmid DNA of SIRT3-Myc and empty vector pcDNA 3.1(+) as a control using Lipofectamine according to the manufacturer’s instructions (Invitrogen, Carlsbad, CA, USA). Lipofectamine-treated cells were used as a mock control (no DNA). After 24 h, the cells were differentiated into adipocytes by the adipocyte differentiation procedure described below.

### 4.3. Adipocyte Differentiation from 3T3-L1 Cells

3T3-L1 preadipocytes (American Type Culture Collection: ATCC, Manassas, VA 20110 USA) were cultured in Dulbecco’s modified Eagle’s medium (DMEM) supplemented with 2.5 ug/mL amphotericin B, 100 I.U./mL penicillin and 100 (μg/mL) streptomycin, 10% fetal bovine serum (FBS), 1mM sodium pyruvate, 2 mM glutamine, and 0.1 mg/mL of primocin into 6 well plates with 2 × 10^5^ cells per well. The following day, after seeding, the cells were differentiated with an adipogenic cocktail made of 1 µM rosiglitazone, 1 µM dexamethasone, 5 µg/mL insulin, and 500 µM 3-isobutyl-1-methylxanthine (IBMX), and treated with 1, 5, 10 µM Honokiol (Sirt 3 activator). Two days after seeding, the cells were differentiated with an adipogenic cocktail made of 1 µM rosiglitazone, 1 µM dexamethasone, 5 µg/mL insulin, and 500 µM 3-isobutyl-1-methylxanthine (IBMX), and were treated with 50 µM and 100 µM 3-TYP (Sirt3 inhibitor). After 2 days, the media was changed to a new DMEM media containing 5µg/mL insulin with the corresponding treatment for 2 additional days, and then the cells were maintained in fresh growth media for another 2–4 day period. At day 6, the cells were terminated for WB and qPCR experiments accordingly. For adipogenesis assessment, at day 8–10, the cells were fixed in 10% phosphate buffered formalin. After fixation, cells were stained with Oil Red O. At different time points, the supernatants were collected for triglyceride (TG) measurement.

### 4.4. Oil Red O Stain

Oil Red O Staining was used for measuring stored lipids of the mature adipocytes from treated cells compared to the control after differentiation. Media was removed from the cells from the 6-well plate and gently washed twice with PBS. For each well, 2 mL of 10% formalin (PERK Scientific, West Chester, PA, USA) was added and incubated for 30 min. Formalin was removed and the cells were gently washed twice with phosphate buffered saline followed by a 5-min incubation in 60% isopropanol. The 60% isopropanol was aspirated and 1 mL of Oil Red O solution (Sigma-Aldrich, St. Louis, MO, USA) was added evenly over the cells in each well. The 6-well plate was rotated and incubated for 10 min. After removing the Oil Red O solution, sterile water was used to wash it four times until excess stain was removed. Images were acquired at 250×, and blinded analyses were performed using ImageJ-analysis software. Oil Red O dye was also eluted with isopropanol and OD was measured at 500 nm for quantification. 

### 4.5. Triglyceride Assay

Cell culture supernatants were collected at certain time points during adipocyte differentiation and triglyceride levels were measured using a colorimetric triglyceride assay kit from Wako (Osaka, Japan). Briefly, supernatants were incubated with reagents from the kit for 5 min at 37 °C and absorbance at 600 nm was measured.

### 4.6. Western Blot

Differentiated adipocytes were harvested and lysed with RIPA buffer (supplemented with proteinase and phosphatase inhibitors). Cell debris was eliminated after centrifugation and clear cell lysate was measured protein concentrations by BCA assay. The proteins (15 µg–30 µg) from cell lysates were run on 10% SDS gel after normalizing the samples with BCA assay. After running the gel, the proteins were transferred to a PVDF membrane, which was then followed by immunoblot analysis with specific antibodies against Sirt 3 (Cat#5490, Cell Signaling Technology, Danvers, MA, USA), PI3K (Cat#4292S, Cell Signaling Technology), pAKT (Cat#13038, Cell Signaling Technology), AKT (Cat# 2920S, Cell Signaling Technology), IR-β (Cat#3025, Cell Signaling Technology), pmTOR (Cat#5536, Cell Signaling Technology), mTOR (Cat#2983, Cell Signaling Technology), pFOXO (Cat#2599, Cell Signaling Technology), pGSK-3β (Cat#5558, Cell Signaling Technology), and GSK-3β (Cat#9832S, Cell Signaling Technology), and β-actin (A1978, Clone AC-15, Sigma) or Tubulin (ab7291, DM1A, Abcam). Secondary antibody anti-rabbit antibody (Cat#7074, Cell Signaling Technology) or anti-mouse anti-body (Cat#7076, Cell Signaling Technology) were used.

### 4.7. RNA Extraction and Real-Time PCR

Cells were harvested, followed by RNA extraction using RNA STAT60 (Tel-Test, Inc., Friendswood, TX, USA) and subsequent purification using PureLink RNA Mini Kit (ThermoFisher Scientific, Waltham, MA, USA). RNA extraction protocol was followed to isolate mRNA. cDNA was synthesized using TaqMan Reverse Transcription reagents (Applied Biosystems, Inc., Foster City, CA, USA) and random hexamer primers according to the recommendations of the manufacturer. Gene amplification using primers (Table 1) was measured with SYBR Green using the CFX Connect™ Real-Time PCR Detection System. Primers detecting for PPAR γ, ACL, SREBP1, C/EBPα, LPL, HSL, ATGL, GLUT4, IL6, resistin, TNF-α, and Sirt3 were used. All reactions were run in triplicate. GAPDH was used as an internal control.

### 4.8. Glucose Uptake

3T3L1 cells were seeded in a 24-well plate at a concentration of 5 × 10^4^ cells per well and differentiated into adipocytes as described in Section 4.3. The cells were treated with HNK at concentrations of 1, 5, and 10 µM or with 3-TYP at concentrations of 50 and 100 µM in stimulation with or without 1 μM insulin. The glucose uptake was performed using Glucose Uptake Colorimetric Assay Kit (Biovision, Catalog # K676) and following manufacturer’s instruction. 

### 4.9. Free Fatty Acid Uptake

3T3L1 cells were seeded in a 6-well plate at a concentration of 2 × 10^5^ cells per well and differentiated into adipocytes as described in Section 4.3. The free fatty acid uptake was performed followed the vendor’s instructions using Fatty Acid Uptake Assay Kit (Biovision, Catalog # K408). Briefly, adipocytes were plated at 50,000 cells/100 μL/well in a 96 well black wall/clear bottom poly-D lysine plate for 5 h, and then the serum was deprived for 1 h. The cells were treated with HNK at concentrations of 1, 5, and 10 µM or with 3-TYP at concentrations of 50 and 100 µM in stimulation with or without 150 nM insulin, and incubated at 37 °C in a 5% CO_2_ incubator for 30 min. At the end of the incubation time, 100 μL of fatty acid mixture was added into the well and incubated for another 60 min; the fluorescence signal was measured with a plate reader using bottom read mode. 

### 4.10. Statistical Analyses

Data from at least three independent experiments were statistically analyzed using a two-tailed unpaired Student’s *t* test or one-way analysis of variance analysis (ANOVA), followed by post hoc Dunnette’s or Tukey’s multiple comparisons or two-way ANOVA with Dunnette’s multiple comparisons by Graphpad prism software (Graphpad Software, La Jolla, CA, USA). 

## 5. Conclusions

In this study, we demonstrated that pharmacologic modulators of Sirt3 Honokiol and 3-TYP have important roles to play in regulating insulin signaling pathway and insulin sensitivity by acting on insulin receptor beta (IR-β) and PI3K/AKT/mTOR pathway, as well as adipogenesis and adipocyte specific cytokines. We identified that Sirt3 activator-induced adipogenesis is associated with increased adipocyte-specific gene expression, especially PPARγ and C/EBPα. Opposed to Sirt3 activator, Sirt3 inhibitor reduced adipogenesis and decreased adipocyte specific gene expression. This reduction of adipogenesis by Sirt3 inhibitor was associated with increased proinflammatory cytokines including IL6, resistin, and TNF, suggesting Sirt3 downregulation might increase the risk of insulin resistance and metabolic abnormalities. We also identified that Sirt3 activation enhanced insulin signaling pathway by activating PI3K/AKT/mTOR cascade and improving free fatty acid and glucose uptake. In contrast, Sirt3 suppression led to decreased insulin signaling pathway and reduced free fatty acid and glucose uptake in 3T3-L1 adipocytes. A future animal model study would be interesting in revealing whether Sirt3 activator works in concert with other physiological processes for improving obesity-induced insulin resistance and other metabolic disease states. We hope our study contributes to the efforts of finding new drug targets and agents for metabolic diseases, including type 2 diabetes, and recommends Sirt3 activators as potential agents in the treatment of insulin resistance and other metabolic abnormalities.

## Figures and Tables

**Table 1:** List of primers used in the study.

Gene	NCBI Ref. Seq. (NM)	Primers	Primer Sequences (5′->3′)
Resistin	NM_022984	F	TCACTTTTCACCTCTGTGGATATGAT
		R	TGCCCCAGGTGGTGTAAA
IL6	NM_001314054	F	CCTCTGGTCTTCTGGAGTACC
		R	ACTCCTTCTGTGACTCCAGC
TNFα	NM_001278601	F	ATGAGCACAGAAAGCATGA
		R	AGTAGACAGAAGAGCGTGGT
ACL	NM_001199296	F	TCCTACAAAGAGGTGGCAGAACT
		R	GGCTTGAACCCCTTCTGGAT
PPAR gamma	NM_001127330	F	TGATTACAAATATGACCTGAAGC
		R	TTGTAGAGCTGGGTCTTTTCAGAAT
SREBP1	NM_011480	F	CACTCCCTCTGATGCTACGG
		R	CTTGTTTGCGATGTCTCCAG
C/EBPα	NM_007408	F	GTTAGCCATGTGGTAGGAGACA
		R	CCCAGCCGTTAGTGAAGAGT
LPL	NM_008509	F	ATGGATGGACGGTAACGGGAATGT
		R	TGGATAATGTTGCTGGGCCCGATA
ATGL	NM_025802	F	GTCCTTCACCATCCGCTTGTT
		R	CTCTTGGCCCTCATCACCAG
HSL	NM_001039507	F	GCAAGATCAAAGCCTCAGCG
		R	GCCATATTGTCTTCTGCGAGTGT
GLUT4	NM_001359114	F	CAGCTCTCAGGCATCAAT
		R	TCTACTAAGAGCACCGAG

## Data Availability

The data that supports the findings of this study are available in the Supplementary Materials of this article.

## Supplementary Materials

The following supporting information can be downloaded at: https://www.mdpi.com/article/10.3390/ijms23073740/s1.

## Abbreviations

Sirt3, Sirtuin-3; Sirt3OE, Sirt3 overexpression; TG, triglyceride; PPARγ, peroxisome proliferator activated receptor gamma; ACL, ATP-citrate lyase; SREBP1, sterol regulatory element- binding protein 1; C/EBPα, CCAAT/enhancer binding protein; qPCR, quantitative polymerase chain reaction; LPL, lipoprotein lipase; ATGL, adipose triglyceride lipase; HSL, hormone-sensitive lipase; GLUT4, glucose transporter 4; LPL, lipoprotein lipase; HSL; ATGL; IL6, interleukin 6; TNF-α, tumor necrosis factor alpha; AKT; pAKT; mTOR, mammalian target of rapamycin; pmTOR; IRβ, insulin receptor beta; FOXO, fork-head box protein; GSK-3β, glycogen synthase kinase-3beta; HNK, honokiol; IGF-1, Insulin-like Growth Factor-1; HNK, honokiol; 3-TYP, (3-(1H-1,2,3-triazol-4-yl) pyridine); SOD, superoxide dismutase; T2DM, type 2 diabetes mellitus; ECM, extracellular matrix; ROS, reactive oxygen species; FBS, fetal bovine serum; IBMX, 3-isobutyl-1-methylxanthine; TG, triglyceride.
