# Supplementary figures and images for: Sirt3 Pharmacologically Promotes Insulin Sensitivity through PI3/AKT/mTOR and Their Downstream Pathway in Adipocytes
Source: Int J Mol Sci. 2022 Mar 29;23(7):3740. doi: 10.3390/ijms23073740 (PMC8998733; doi:10.3390/ijms23073740)

Control

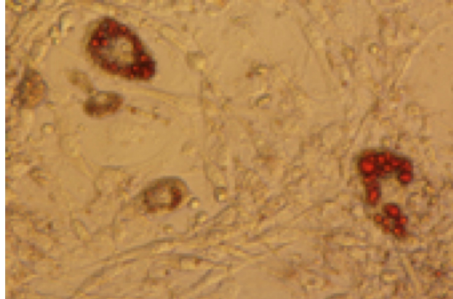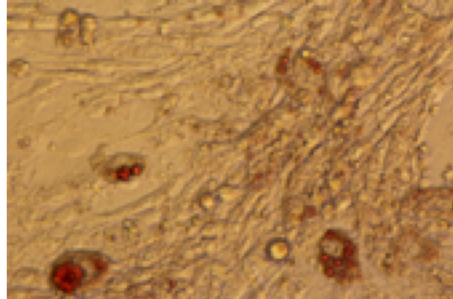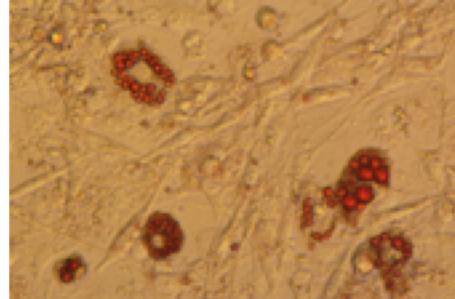

HNK\_1 $\mu$ M

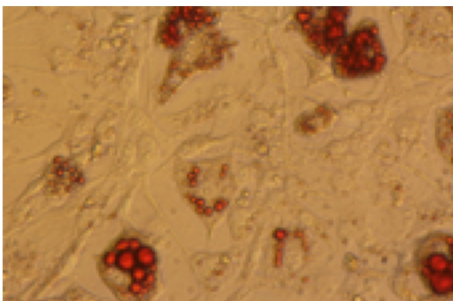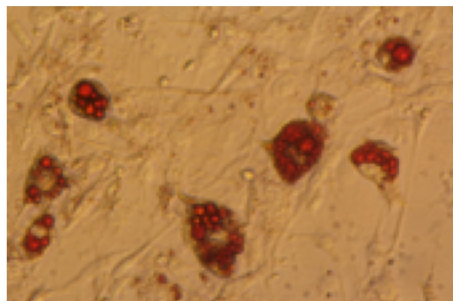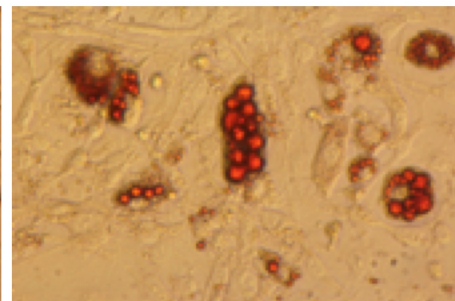

HNK\_5 $\mu$ M

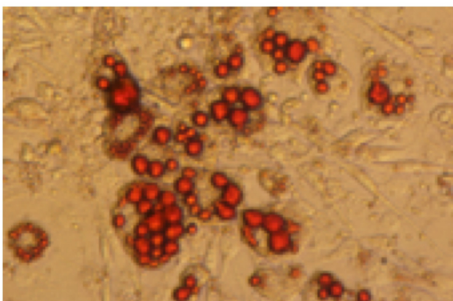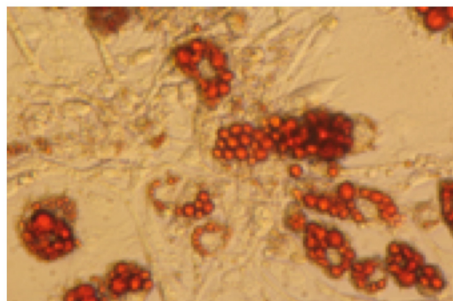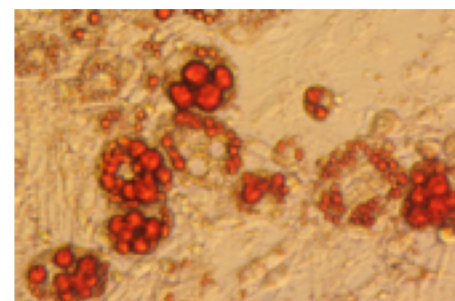

HNK\_10 $\mu$ M

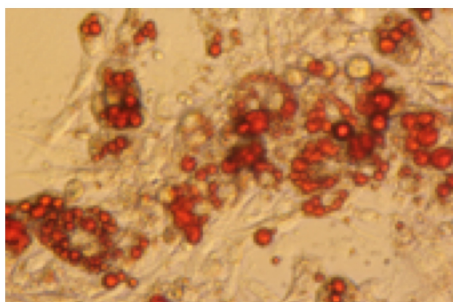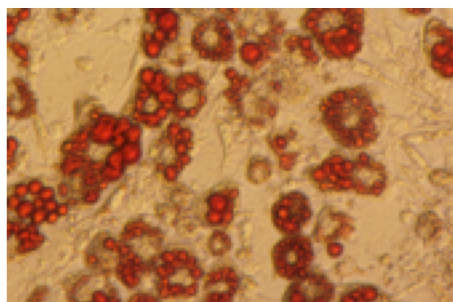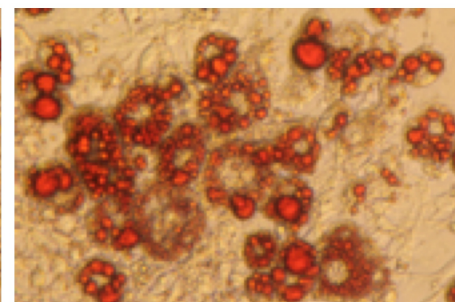

Control

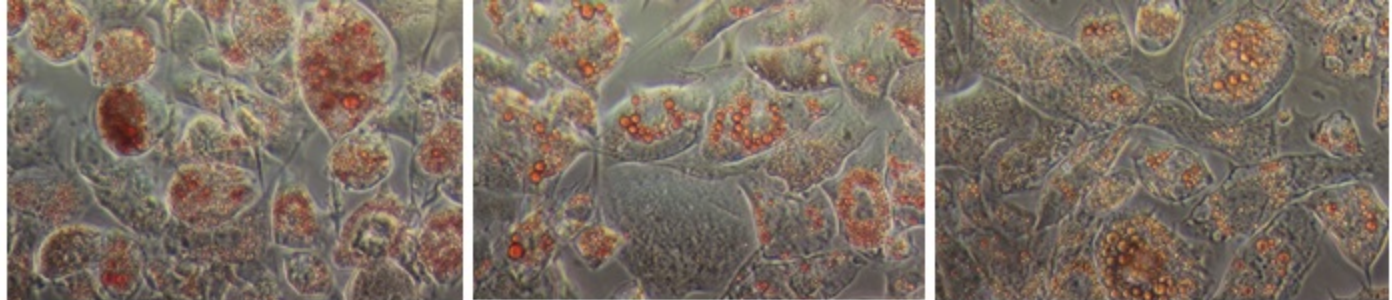

3-TYP\_50 $\mu$ M

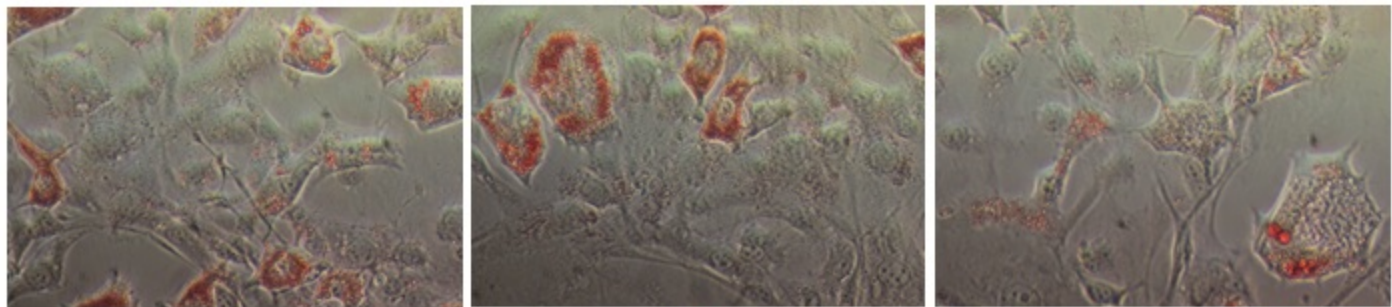

3-TYP\_100 $\mu$ M

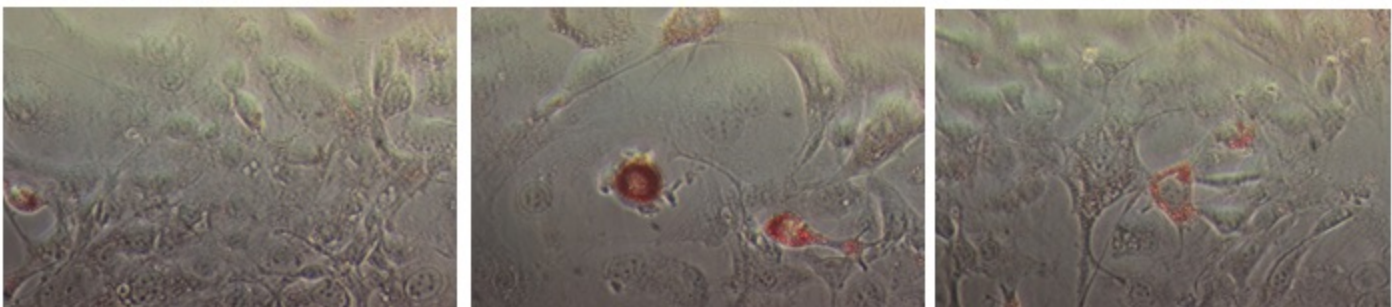

Day 1

Day 2

Day 3

Day 4

CTL

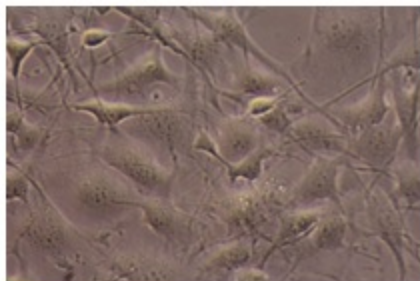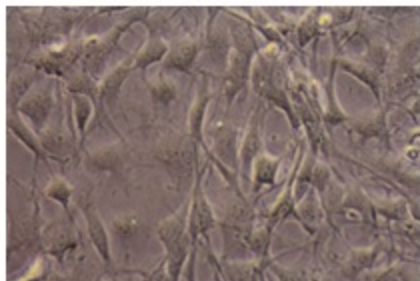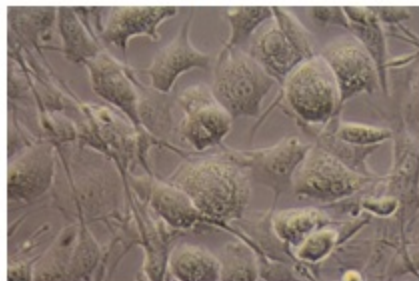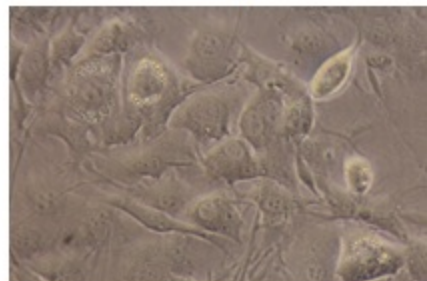

Sirt3  
induction

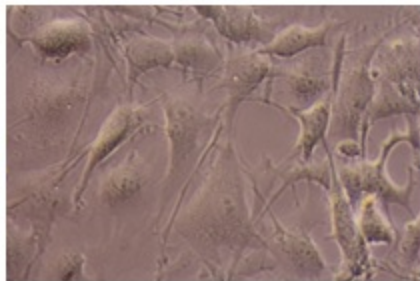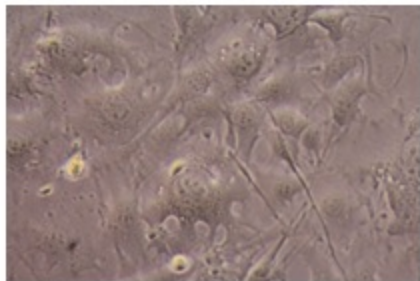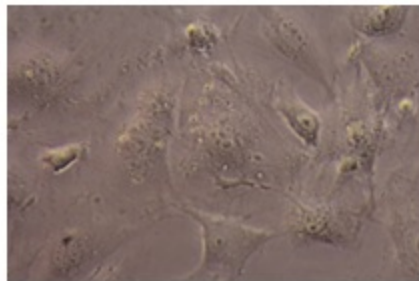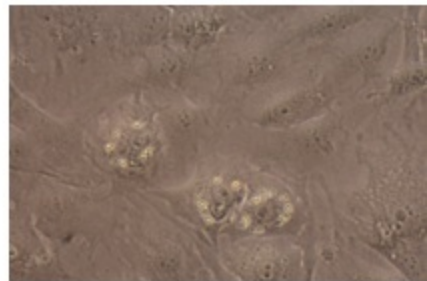

Supplement: Supplementary file 1 [file ijms-23-03740-s001.zip › ijms-1610434-supplementary.pdf]
